# Function and Localization of the *Arabidopsis thaliana* Diacylglycerol Acyltransferase DGAT2 Expressed in Yeast

**DOI:** 10.1371/journal.pone.0092237

**Published:** 2014-03-24

**Authors:** Laure Aymé, Sébastien Baud, Bertrand Dubreucq, Florent Joffre, Thierry Chardot

**Affiliations:** 1 INRA, UMR1318, Institut Jean-Pierre Bourgin, Saclay Plant Sciences, Versailles, France; 2 AgroParisTech, Institut Jean-Pierre Bourgin, Saclay Plant Sciences, Versailles, France; 3 ITERG, Pessac, France; University of Graz, Austria

## Abstract

Diacylglycerol acyltransferases (DGATs) catalyze the final and only committed step of triacylglycerol synthesis. DGAT activity is rate limiting for triacylglycerol accumulation in mammals, plants and microbes. DGATs belong to three different evolutionary classes. In *Arabidopsis thaliana*, DGAT1, encoded by At2g19450, is the major DGAT enzyme involved in triacylglycerol accumulation in seeds. Until recently, the function of DGAT2 (At3g51520) has remained elusive. Previous attempts to characterize its enzymatic function by heterologous expression in yeast were unsuccessful. In the present report we demonstrate that expression of a codon-optimized version of the *DGAT2* gene is able to restore neutral lipid accumulation in the *Saccharomyces cerevisiae* mutant strain (H1246), which is defective in triacylglycerol biosynthesis. Heterologous expression of codon-optimized *DGAT2* and *DGAT1* induced the biogenesis of subcellular lipid droplets containing triacylglycerols and squalene. Both DGAT proteins were found to be associated with these lipid droplets. The fatty acid composition was affected by the nature of the acyltransferase expressed. DGAT2 preferentially incorporated C16:1 fatty acids whereas DGAT1 displayed preference for C16:0, strongly suggesting that these enzymes have contrasting substrate specificities.

## Introduction

Triacylglycerols (TAGs) are the main form of fatty acid (FA) storage in most eukaryotes [1]. They accumulate in dedicated organelles called lipid droplets (LDs). LDs are composed of a neutral lipid core surrounded by a monolayer of phospholipids (PLs), in which various types of specialized proteins are embedded. LDs serve as an energy reservoir, used for example during seed germination in plants [2], and are the source of signaling molecules and substrates for membrane biogenesis [1].

Three enzymes catalyze TAG synthesis by the acylation of *sn*-1,2-diacylglycerol (DAG) [3]. Two acyltransferases catalyze acyl-CoA independent reactions. Phospholipid: diacylglycerol acyltransferase (PDAT, EC 2.3.1.158) uses PLs as acyl donors. Its activity has been reported in yeast and plants and corresponding genes have been identified [4], [5]. DAG transacylase uses two DAGs as substrates to produce one TAG and one monoacylglycerol (MAG). This activity has been reported in various organisms [6], [7] but to date, genes encoding this enzyme remain unknown. In contrast to PDAT and DAG transacylase, acyl-CoA: diacylglycerol acyltransferases (DGAT, EC 2.3.1.20) are acyl-CoA dependent enzymes, which catalyze the final and only committed step of the Kennedy pathway [8], [9], [10]. In seeds, the absence of DGAT activity leads to a significant reduction in TAG accumulation [11], [12].

DGAT genes fall within three distinct families that share no sequence homology and probably result from convergent evolution [3], [13]. DGAT1 belongs to the superfamily of membrane bound O-acyltransferases [14], which includes acyl-CoA:cholesterol acyltransferase-1 and -2 (ACAT, EC 2.3.1.26). DGAT2 belongs to the same family as acyl-CoA:monoacylglycerol acyltransferases (EC 2.3.1.22) and wax monoester synthases (2.3.1.75) [15]. The existence of a cytosolic form of DGAT, belonging to a third family was reported in developing peanut cotyledons [16] and in *Arabidopsis thaliana* seedlings [17].

DGAT1 and 2 are found in mammals. These endoplasmic reticulum (ER) integral proteins [18], [19] are both responsible for TAG production [20], [21] and presumably have different physiological functions [22], [23], [24]. The most significant contribution to TAG synthesis in the yeast *Saccharomyces cerevisiae* is from Dga1p, a member of the DGAT2 family [25], [26]. In the oleaginous yeast *Yarrowia lipolytica* Dga2p, a member of the DGAT1 family makes a major contribution to TAG synthesis [9].

In *A. thaliana* DGAT1 encoded by At2g19450 plays a major role in seed lipid accumulation. Its activity and function have been extensively characterized *in planta* [8], [27]. DGAT1 inactivation results in a 20 to 40% decrease in seed oil content [12], [28], [29]. The observation that oil deposition was not completely abolished in this mutant background strongly suggested that other enzymes also contribute to TAG accumulation in seeds. Accordingly, a concomitant down regulation of PDAT1 and DGAT1 further decreased the seed oil content (by 70 to 80%), thus establishing that PDAT1 and DGAT1 play partially redundant functions in seeds [28].

DGAT2 coding sequences are found in the genome of several plants. This enzyme can incorporate unusual FAs into TAGs [30], such as eleostearic acid, a polyunsaturated FA found in Tung tree (*Vernicia. fordii*) [31], or ricinoleic acid, a hydroxy FA from *Ricinus communis* [32]. The *A. thaliana* genome encodes a putative DGAT2 (At3g51520), the functionality of which was recently demonstrated by transient expression in *Nicotiana benthamiana* leaves [33]. However, seeds from *A. thaliana dgat2* mutants do not show altered TAG accumulation and introduction of the *dgat2* mutation in a *dgat1* mutant background did not aggravate the *dgat1* seed phenotype, suggesting that DGAT2 is not involved in seed oil accumulation [28].

The current model of LD biogenesis proposes that LDs occur in specialized subdomains of the ER, gathering enzymes of the neutral lipid biosynthetic pathway [1]. Accumulation of lipids between the two membrane leaflets would lead to budding of LDs covered with a PL monolayer [1], [34]. The presence of proteins normally associated with the ER membrane at the surface of LDs is in favor of the budding model. This has been verified for *A. thaliana* DGAT1 [27] and DGAT2 [35].

Here we report the functional characterization of *A. thaliana* DGAT2 in the yeast mutant strain H1246 which is unable to accumulate neutral lipids [26]. We demonstrate that yeast codon usage limits protein expression in this system and that expression of codon-optimized DGAT2 can restore TAG synthesis by functional complementation. *A. thaliana* DGAT1 was used as a positive control. We then show that the two DGAT proteins are associated with LDs. Both enzymes exhibit contrasted substrate specificity and induce squalene accumulation in LDs.

## Materials and Methods

### Cloning of DGAT sequences for expression in yeast

The *A. thaliana DGAT1* (At2g19450) and *DGAT2* (At3g51520) cDNAs (Fig. S1) were amplified with proofreading Pfu Ultra DNA polymerase (STRATAGENE, La Jolla, CA, USA) from a mixture of seed cDNAs (of the Ws ecotype) using the primers: 5′-*att*B1-ATGGCGATTTTGGATTC-3′ and 5′-*att*B2-TCATGACATCGATCCTTTTC-3′ (*DGAT1*); 5′-*att*B1-ATGGGTGGTTCCAGAG-3′ and 5′-*att*B2-TCAAAGAATTTTCAGCTCAAG-3′ (*DGAT2*). *Att*B1 and *att*B2 refer to the corresponding Gateway recombination sequences. BP recombination was used to introduce the PCR products into the *pDONR207* entry vector (INVITROGEN, Carlsbad, CA, USA) for sequencing. Codon sequences were optimized for expression in *S. cerevisiae* (Fig. S1) by Eurofin MWG Operon (Ebersberg, Germany). These sequences were amplified using Phusion Hot Start polymerase (Thermo Scientific, Illkirch, France) and introduced into the BamHI/SmaI restriction sites of the *pRT21* vector (also known as *pNBT29*) [36] to generate proteins fused with a C-terminal GFP. Corresponding constructs were made with a stop codon introduced for expression of DGAT alone without the GFP fusion. Details of the oligonucleotides used are shown in **Table 1**. All constructs were sequenced to confirm the absence of mutations in amplified sequences (Genoscreen, Lille, France).

**Table 1:** Oligonucleotide primers used in this study.

Gene	Primer sequences (restriction sites are underlined)	Features
*DGAT1*		
*At2g19450F*	5′ GGGATCCATGGCGATTTTGGATTCTGCTG 3′	BamHI
*At2g19450optF*	5′ CGGGATCCATGGCCATCTTAGACTC 3′	BamHI
*At2g19450R*	5′ CCCCCCGGGTGACATCGATCCTTTTCGGTTC 3′	SmaI, no stop
*At2g19450optR*	5′ CCCCCCGGGCTAGGACATAG 3′	SmaI
*DGAT2*		
*At3g51520F*	5′ CGGGATCCATGGGTGGTTCCAGAGAGTTC 3′	BamHI
*At3g51520optF1*	5′ CGGGATCCATGGGTGGTTCTAGAG 3′	BamHI
*At2g19450R*	5′CCCCCCGGGAAGAATTTTCAGCTCAAGATCATAGC 3′	SmaI, no stop
*At3g51520optR*	5′ CCCCCCGGGCTATAGTATCTTCAG 3′	SmaI

Opt: oligonucleotides designed for amplification of optimized genes

### Expression of DGATs in yeast

Details of the recombinant plasmids as well as an empty plasmid control used to transform the *S. cerevisiae* quadruple mutant (*are1*, *are2*, *dga1*, *lro1*) strain H1246 or the SCY62 wild-type (WT) strain [26] are given in **Table 2**. Cells were grown in Ura deficient medium as described in [37] and protein expression was induced with 2% galactose in the presence of 0.02% glucose.

**Table 2:** Plasmids used in this study.

Plasmids	Host strain	Source/ref
*pRT21* also known as *pNBT29*	H1246, SCY62	Froissard *et al.*, 2006 [36]
*pRT21-DGAT1-GFP*	H1246	This work
*pRT21-DGAT2-GFP*	H1246	This work
*pRT21-DGAT1_opt_-GFP*	H1246	This work
*pRT21-DGAT2_opt_-GFP*	H1246	This work
*pRT21-DGAT1_opt_*	H1246	This work
*pRT21-DGAT2_opt_*	H1246	This work

### Lipid droplet purification

Yeast LDs were separated by sucrose density gradients as previously described by Yu *et al.* [38]. Cells corresponding to 400 UA_600nm_ were harvested by centrifugation, washed with water and resuspended in Fat Body Buffer (FBB, 10 mM HEPES, 10 mM KCl, 0.1 mM EDTA, 0.1 mM EGTA, pH 7.5) supplemented with protease inhibitors (cOmplete, Mini, EDTA-free, Roche, Indianapolis, USA). Cells were disrupted with a One Shot cell disruptor (Constant Systems Ltd, Daventry, UK) at a pressure of 2.97 kbar. Lysates were centrifuged for 10 min at 12 000 g. The supernatant volume was adjusted to 5.4 ml with FBB containing sucrose at a final concentration of 0.54 M. It was overlaid sequentially with FBB containing 0.27 M, 0.135 M and 0 M sucrose (3×1.8 mL). The gradients were subjected to ultracentrifugation for 90 min at 150 000 g and 4°C in a SW41 Ti swing-out rotor (Beckman Coulter, Villepinte, France). The floating lipid layer, or the corresponding volume for the strain transformed with the empty plasmid, was collected and stored at −80°C. The hydrodynamic diameter of purified LDs was assessed by dynamic light scattering on a Malvern HPPS (Malvern Instruments Ltd, Worcestershire, UK) according to Vindigni *et al*. [39].

### SDS-PAGE and immunoblotting

LD proteins were separated using 10% ready-to-use NuPAGE gels and LDS sample buffer (Invitrogen, Cergy Pontoise, France). Proteins were transferred to PVDF membrane (Immobilon-P, 0,45 μm pore size, Merck-Millipore, Billerica, USA) and proteins fused to GFP were probed with a mouse anti-GFP IgG (dilution 1∶1000) (Roche, Indianapolis, USA). Primary antibody was detected using horseradish peroxidase-conjugated anti-mouse IgG secondary antibody (dilution 1∶2500) (Sigma-Aldrich, L’Isle d’Abeau, France), and revealed using SuperSignal West Dura Extended Duration Substrate (Thermo Scientific, Illkirch, France). Luminescence was recorded using the LAS-3000 imaging system and MultiGauge software (Fujifilm, Saint Quentin en Yvelines, France).

### Lipid extraction

Lipids from yeast cells or LDs were extracted as described by Folch *et al.* [40]. Cells were harvested by centrifugation and washed twice with a solution containing 0.5% BSA and 0.9% NaCl. After freeze-drying, 100 mg of dry cells or LDs purified from 1000 UA_600nm_ were resuspended in 5 ml of chloroform/methanol 2/1 (v/v). After 1h incubation with shaking, the extract was centrifuged for 5 min at 900 g and the supernatant was recovered and mixed with 2.5 ml of 0.9% NaCl. The organic phase was collected after centrifugation at 900 g for 5 min and washed three times with chloroform/methanol/water 3/48/47 (v/v/v) [41]. Solvents were evaporated under a stream of nitrogen.

### Lipid analysis

Lipid classes were separated by thin-layer chromatography (TLC). Lipids were solubilized in chloroform/methanol 2/1 (v/v) before separation on silica-coated aluminum plates (TLC Silica gel 60 F_254_, Merck, Fontenay Sous Bois, France) pre-washed in chloroform/methanol 1/1 (v/v). Plates were developed twice with petroleum ether/diethyl ether/acetic acid 50/50/2 (v/v/v) and petroleum ether/diethyl ether 49/1 (v/v) to 5 and 12 cm [42], [43]. Lipids were stained with 5% phosphomolybdic acid for 30 min at 100°C. Lipids were identified based on the migration of lipid standards (Sigma-Aldrich, L’Isle d’Abeau, France). Plates were scanned using an Epson expression 1680 Pro Scanner. When necessary, lipids were quantified by densitometry using MultiGauge software (Fujifilm), according to Spanova *et al.* [44].

### Fatty acid determination

#### Total lipids

The FA content and composition of yeast strains were determined according to Browse *et al.* [45]. After freeze-drying, 20 mg of dry yeast were incubated in 2 ml of MeOH/H_2_SO_4_ (100/2.5, v/v) at 80°C for 90 min after addition of heptadecanoic acid as an internal standard. After addition of 900 μl of hexane and 3 ml of 0.9% NaCl, FA Methyl Esters (FAMEs) were extracted into the organic phase and analyzed by gas chromatography with flame ionization detection (GC-FID) according to Froissard *et al.* [37].

#### Neutral lipids

Neutral lipids were obtained upon fractionation of total lipids using an Isolute SPE Aminopropyl column (ALLTECH France Sarl, Epernon, France) according to [46]. After transmethylation, FAMEs were identified and quantified by GC-FID.

### Compositional analysis and quantification of the neutral lipid fraction

Neutral lipids were derivatized to TriMethylSylil-lipids (TMS-lipids) prior to their analyses by GC-FID using 100 μL of reagent solution (n-methyl imidazole/N-methyl-N-trimethylsilyl-heptafluorobutyramide, 1/20, v/v). Analysis of TMS-lipid derivatives were performed on a Trace GC Ultra gas Chromatograph (Thermo Scientific, Illkirch, France) equipped with a fused-silica ZB5 HT capillary column (15 m×0.25 mm I.D., 0.1μm film thickness; Phenomenex, Le Pecq, France). Samples were injected into an on-column detector and the FID system was set at 400°C. The oven temperature program was 100°C, increased to 370°C at 10°C/min and isothermal for 5 min at this final temperature (total run time 32 min). The carrier gas (H_2_) flow was maintained constant at 1.5 mL/min. A mixture containing the following compounds: FAs (C16, C18 and C18:1), MAG (C16, C18 and C18:1), DAG (equivalent to 34 and 36 atoms of carbon), TAG (equivalent to 48, 50, 52 and 54 atoms of carbon), squalene, cholesterol and phytosterols (campesterol, stigmasterol and sitosterol) was used as the standard for further product identification [47].

### Squalene identification

After extraction, 100 μg of lipids were resuspended in 300 μl hexane and dried over Na_2_SO_4_. Silylation was performed on 10 μl of the sample with 50 μl of BSTFA and 5 μl of pyridine (Sigma-Aldrich, L'Isle d'Abeau, France). Lipids were separated on a DB1 supelco capillary column of 15 m×0.32 mm (carrier gas: helium at a constant flow rate of 1.5 ml.min^−1^) with a first increase from 45°C to 180°C (at 30°C.min^−1^) and a second rise from 180°C to 280°C (at 3°C.min^−1^). Detection was performed by mass spectrometry on a Varian 4000 ion trap operating in the electron impact mode (70 eV) with ions detected on a range of 50 to 800 *m/z*. The presence of squalene in the sample was assessed by comparing the mass spectra of the component having an identical retention time to squalene with the mass spectra of squalene (≥ 98% squalene, Sigma-Aldrich)

### Confocal Image acquisition

Yeast were incubated for 20 min in a Nile Red solution (1 g/l from a 1 g/ml stock solution in acetone), and washed with PBS solution containing 10% v/v glycerol. Images were acquired using an inverted LEICA SP2-AOBS spectral confocal laser microscope (LEICA Microsystems, Mannheim, Germany) using an HCX PL APO 63 X water (long distance) 1.2 objective. GFP and Nile Red fluorescence were observed with a 488 nm light wavelength generated by an argon laser and an emission band of 500−520 nm and 600−650 nm respectively. Transmitted light was captured with a dedicated photomultiplier (PMT trans). Each image is the average of eight scans at a resolution of 512×512 pixels with 8x numerical zoom.

## Results

### Codon usage affects expression of DGAT2 and DGAT1 in *S. cerevisiae*

The DGAT2 (At3g51520) sequence is highly similar to several active DGAT2 genes from plants [31], [32]. However, inactivation of the DGAT2 gene did not significantly reduce seed oil content [28] and expression of the gene in *S. cerevisiae* did not lead to lipid accumulation [28], [48], [49], [50]. Taken together, these findings firstly suggested that DGAT2 does not encode a functional DGAT protein [28]. Nevertheless, a very recent report has shown that DGAT2 can be actively expressed in *N. benthamiana* cells [33] and exhibits a higher specificity for C18:3 FA.

**Figure 1:**
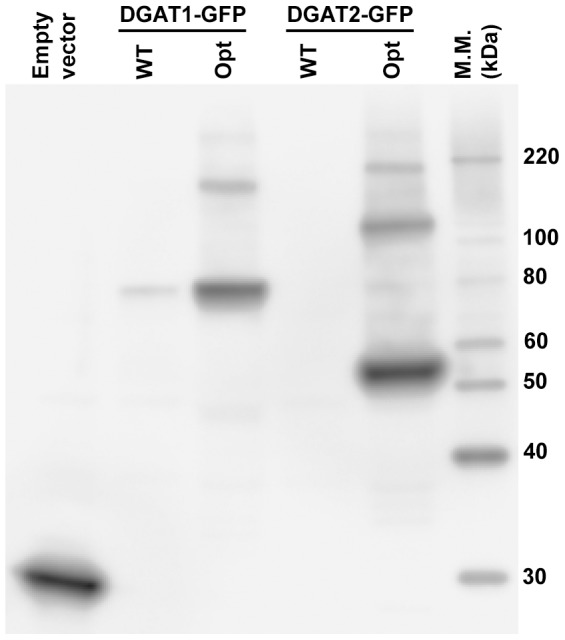
Effect of codon usage on the expression of DGATs. H1246 strains expressing GFP alone (empty vector), wild-type (WT) or an optimized (opt) version of DGAT-GFP were induced for 18 h. Yeast protein extracts corresponding to 0.06 UA_600nm_ were separated by SDS-PAGE and transferred to a PVDF membrane. The membrane was incubated with anti-GFP antibody, and revealed with an anti-IgG-HRP secondary antibody. Respective molecular masses of DGAT1_opt_-GFP and DGAT2_opt_-GFP are 86 and 63 kDa. The marker used was MagicMark (Invitrogen).

The function of *A. thaliana* DGAT2 was examined by heterologous expression in the H1246 *S. cerevisiae* strain. This strain lacks four acyltransferases and is completely defective in neutral lipid biosynthesis [26]. Codon usage can affect expression of heterologous proteins in *S. cerevisiae* [3], [51], thus leading to the false conclusion that the expressed proteins are not active. Liu *et al*. [3] suggested that the lack of DGAT2 activity observed in *S. cerevisiae* could be due to differences in codon usage between this yeast and *A. thaliana*. To test this hypothesis and allow detection of the expressed proteins, we used WT or optimized versions of sequences encoding *A. thaliana* DGAT1 and 2 and harboring GFP tags for expression in *S. cerevisiae*. Accumulation of the two optimized DGATs in total yeast extracts was confirmed by Western blot analysis with anti-GFP antibodies (**Fig. 1**). When codon bias was not considered only slight expression of DGAT1 was observed, confirming previous results [27] and demonstrating the importance of codon optimization for plant DGAT expression in yeast. High molecular mass proteins were identified by immunoblotting which may reflect DGAT oligomerization as previously suggested for DGATs from other organisms [15], [18], [19], [52].

### DGAT2 restores neutral lipid accumulation in H1246

**Figure 2:**
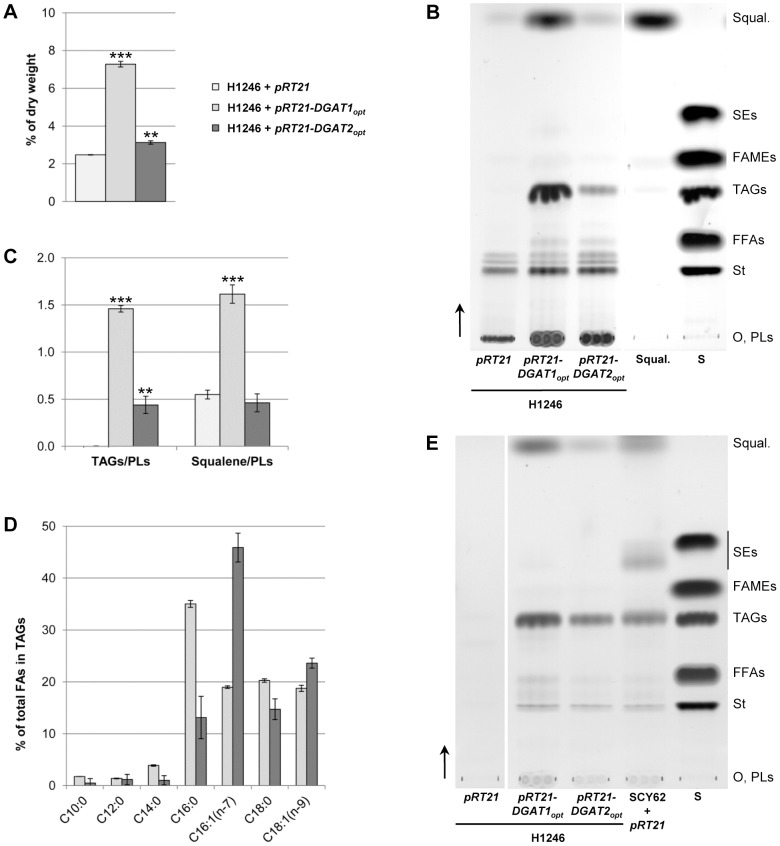
Lipid analysis of the H1246 strain expressing DGATs. H1246 strains transformed with the empty vector or expressing DGAT1_opt_ or DGAT2_opt_ were induced for 18 h. (**A**) **FA content of yeast lipids.** FAs from 20 mg of dry yeast were transmethylated. The resulting FAMEs were identified and quantified by GC-FID. Values are expressed in % of dry weight. Asterisks indicate statistically significant differences according to *t*-test (**P<0.01; ***P<0.001). (**B**) **Separation of yeast lipids by TLC.** Lipids were extracted from 100 mg of dry yeast. Lipids from 2 mg dry yeast were separated on a silica plate. This experiment is representative of three independent cultures. Vertical arrows indicate the direction of migration. (**C**) **TAG and squalene contents.** Relative amounts of TAGs and squalene to PLs were evaluated by densitometry after separation of yeast lipids by TLC (Fig. 2B). Values were calculated from three independent cultures. Asterisks indicate statistically significant differences according to a *t*-test (**P<0.01; ***P<0.001). (**D**) **FA composition of yeast TAGs.** Lipids were extracted from 100 mg of dry yeast. Neutral lipids were separated by SPE and transmethylated. The resulting FAMEs were identified and quantified by GC-FID. Values are expressed in % of total FAs in TAGs. (**E**) **Separation of LD lipids by TLC.** Lipids extracted from purified LDs of 30 UA_600 nm_ from recombinant H1246 strains or from the WT strain (SCY62) transformed with the empty vector were separated on silica plates. Vertical arrows indicate the direction of migration. O: origin of migration; S: standard; PLs: phospholipids, St: sterols; FFAs: free fatty acids; TAGs: triacylglycerols; FAMEs: fatty acid methyl esters; SEs: sterol esters, Squal: squalene.

To study the effect of DGAT2 expression on the H1246 lipid profile, the quadruple mutant was transformed with an optimized version of the protein with no GFP tag (DGAT2_opt_). As a positive control, we used an H1246 strain transformed with an optimized version of DGAT1 (DGAT1_opt_). Total FAs from the different strains were quantified following transmethylation and GC-FID analysis. Expression of both DGAT1_opt_ and DGAT2_opt_ significantly increased total FA content in H1246 (**Fig. 2** **.A**). Cells expressing DGAT2_opt_ and DGAT1_opt_ contained 1.3 and 2.9 times more FAs, respectively, than H1246 transformed with an empty vector (negative control). Total lipids from the recombinant yeast were extracted and separated on TLC plates **(** **Fig. 2** **.B)**. Extracts purified from both DGAT expressing strains contained TAGs. Densitometry showed that the DGAT2_opt_ expressing strain contained 3.3 times less TAGs than the DGAT1_opt_ expressing strain (**Fig. 2** **.C**). In addition, a lipid which accumulated in both these DGAT expressing lines had a retention factor identical to that of squalene (**Fig. 2** **.B**). Subsequent GC-MS analysis confirmed the identity of the product (**Fig. S2**). The highest squalene accumulation, determined by densitometric comparison of various amounts of squalene, reached approximately 2.7 g/kg in the DGAT1_opt_ expressing strain.

FA composition analyses of the neutral lipid fraction (TAGs) by GC-FID revealed differences depending on expression of DGAT1_opt_ or DGAT2_opt_. The DGAT2_opt_ expressing strain (**Fig. 2** **.D**) accumulated mostly C16:1 whereas the DGAT1_opt_ expressing strain preferentially accumulated C16:0. However, no significant differences were found for the TAG fraction (**Fig. S3**). TAG profiles of both DGAT1_opt_ and DGAT2_opt_ expressing cells showed a normal distribution centered on C50, close to 35% with also a high proportion of C48 (18%) and C52 (23%). A small proportion of C54 was also noted.

### Expression of DGAT2 and DGAT1 in *S. cerevisiae* H1246 restores LD biogenesis

No LDs form in the H1246 mutant strain [26]. Neutral lipid production was re-established in yeast expressing optimized sequences encoding DGAT2 or DGAT1 as well as the non-optimized sequence coding for DGAT1 (data not shown). We attempted to purify LDs from H1246 transformed with the empty *pRT21* vector or *pRT21-DGAT1_opt_* or *pRT21-DGAT2_opt_*. No floating layer was recovered from the H1246 strain transformed with the empty vector. Using dynamic light scattering we determined that the floating layers recovered from DGAT1_opt_ expressing cells contained objects with diameters similar to LDs from a WT strain (SCY62, 330nm). In cells expressing DGAT2_opt_ we observed smaller objects (220 nm).

Lipids contained in the floating layers were extracted and separated by TLC (**Fig. 2** **.E**). Lipids from DGAT1_opt_ and DGAT2_opt_ expressing cells were mainly TAGs, squalene and sterols, but lacked sterol esters in contrast to lipids extracted from the WT strain. No lipids could be extracted from the top fraction of the gradients for the H1246 strain transformed with the empty vector.

### DGAT2 and DGAT1 colocalize with intracellular neutral lipids

**Figure 3:**
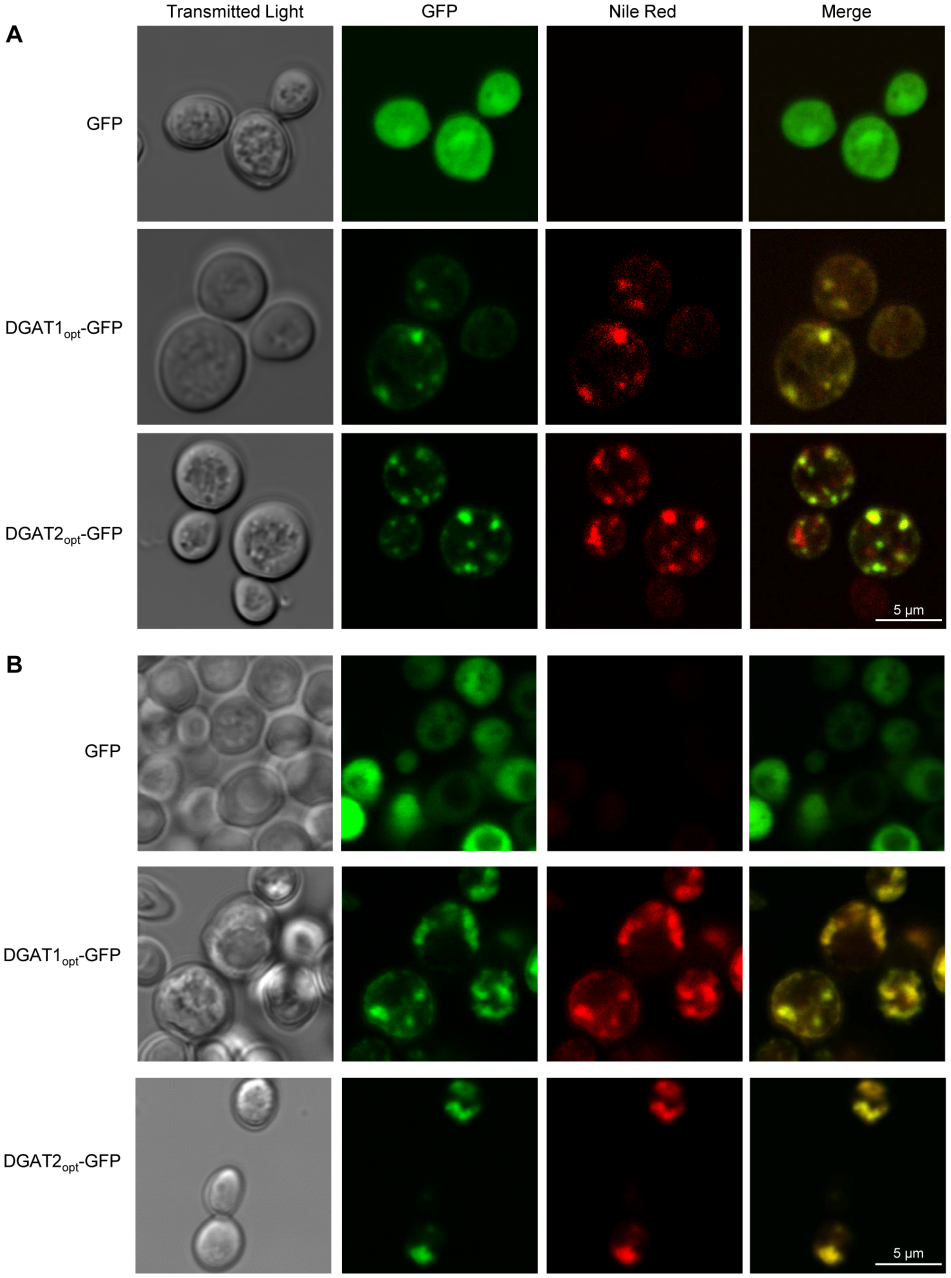
Subcellular localization of DGATs and neutral lipids in yeast by confocal microscopy. H1246 strains expressing GFP alone, DGAT1_opt_-GFP or DGAT2_opt_-GFP were induced for 8 h (**A**) and 18 h (**B**) and observed with a confocal microscope, after incubation with Nile Red. GFP and Nile Red fluorescence was excited with a 488 nm light wavelength generated by an argon laser. Emitted light was collected at 500−520 nm (GFP) and 600−650 nm (Nile Red).

Confocal fluorescence microscopy of DGAT1_opt_-GFP and DGAT2_opt_-GFP revealed the presence of the proteins in typical lipid particle structures after 8 h induction (**Fig. 3** **.A**). The same staining pattern was observed using the lipophilic fluorescent dye Nile Red which selectively accumulated in LDs (**Fig. 3** **.A**), thus confirming the localization of both DGAT proteins in this compartment. Similar results were obtained after 18 h induction, but neither GFP nor Nile Red were found in punctuate structures (**Fig. 3** **.B**).

## Discussion

In *A. thaliana*, DGAT2 inactivation did not modify seed oil content. Furthermore, mutation of DGAT2 in the DGAT1 mutant background did not lead to any additional phenotypes, leading Zhang *et al.* [28] to conclude that DGAT2 does not play a substantial role in seed lipid accumulation. The possibility that DGAT2 could be expressed and active in vegetative tissues was very recently demonstrated by Zhou [33]. Despite the fact that *A. thaliana* DGAT2 shares 71% and 58% identity with active Tung tree [31] and *R. communis* [32] DGAT2, previous heterologous expression experiments did not lead to TAG accumulation in the yeast mutant strain H1246 [28], [48]. Liu *et al*. [3] suggested that the *A. thaliana DGAT2* gene may exhibit a strong codon bias affecting its expression in yeast. In this report, we demonstrated that using yeast codon usage is mandatory for DGAT2 expression (**Fig. 1**) and to restore TAG accumulation (**Fig. 2** **.B**) in H1246. Optimization of *A. thaliana* DGAT1 codon usage also significantly increased the rate of protein synthesis and enhanced TAG accumulation. Heterologous DGAT expression is thus controlled by subtle usage of tRNA via codon bias.

H1246 expressing DGAT1 accumulated FA levels of up to 7% of dry weight (**Fig. 2** **.A**). We also found a significant amount of squalene in this strain (**Fig. 2** **.B**). Squalene is a triterpene precursor in sterol biosynthesis with potential uses in nutrition, cosmetics, and medicine [53]. Up to 2.7 g of squalene per kg of dry biomass were produced in this strain. This value, obtained in non-optimized conditions, is comparable to the reported yields of promising bioindustrial strains [53]. Spanova *et al*. [54] showed that the LD is a neutral lipid depository able to accommodate squalene. The WT yeast strain (SCY62) naturally accumulates squalene in LDs in addition to TAGs and sterols esters (**Fig. 2** **.E**). We found small amounts of squalene in the H1246 strain (**Fig. 2** **.B**) even though it lacks LDs [26] (**Fig. 2** **.E**). These results are consistent with those of Spanova *et al*. [54] who demonstrated that in the absence of LDs, in a *dga1*Δ*lro1*Δ*are1*Δ*are2*Δ mutant strain, squalene can be stored in microsomal and mitochondrial membranes. In contrast to DGAT1, however, LD biosynthesis was probably not high enough in DGAT2-expressing cells for significant amounts of squalene to be stored. The amount of sterols found in all cells and LDs (**Fig. 2** **.B and 2.E**) did not vary, strongly suggesting that sterol biosynthesis is uncoupled from the level of its precursor squalene.

C16 and C18 FAs were the major FA species acylated in TAGs from both DGAT expressing strains (**Fig. 2D**), DGAT2 preferred C16:1 whereas DGAT1 exhibited specificity toward C16:0. The method used for TAG profiling does not differentiate TAG containing saturated from unsaturated FAs [47], thus the results could not highlight differences between neutral lipids from strains expressing DGATs (**Fig S3**). Depending on the plant species, DGAT1 or DGAT2 is a major enzyme responsible for the accumulation of seed TAG. *A. thaliana* DGAT1 is involved in acylation of the *sn*-3 position of the glycerol. Stereochemical analysis showed that the *sn*-1 and *sn*-3 positions are usually saturated, while unsaturated species occur mainly at *sn*-2 [55]. In castor bean and Tung tree, DGAT2 is specific for unsaturated FAs [31], [32]. The specialization of DGAT2 (C16:1) observed in the present study is consistent with these last findings. Similarly, transient heterologous expression of DGAT2 in tobacco leaves leads to the accumulation of TAG containing unsaturated FA [33] revealing possible expression under the subtle control of tissue specific factors. Thus, very long chain FA or polyunsaturated FA are potential acyl donors found in *A. thaliana* seeds and *N. benthamiana* leaves, but absent in *S. cerevisiae*. It is therefore difficult to compare enzyme specificity and velocity as reflected by lipid accumulation for DGATs expressed in heterologous systems. Overall, the results strongly suggest that these enzymes show specificity toward different acyl donors.

Our confocal microscopy investigations confirmed the co-localization of DGAT-GFP and neutral lipids. The punctuate structures observed after a short induction (8 h, **Fig. 3** **.A**) were typical of LDs, however Nile Red staining was more diffuse after longer induction times (18 h, **Fig. 3** **.B**). The lipids which accumulated in DGAT expressing yeast were different from the WT strain and lacked sterol esters. As previously suggested by Czabany [56], lipid composition affects LD protein content and as a consequence, the interactions between LDs and their environment could be modified. Nevertheless the objects purified from the yeast which had been induced for 18 h still had a typical LD hydrodynamic diameter. Thus it could be that close association of numerous LDs rendered individual droplets undistinguishable *in vivo* using light microscopy, explaining the shape of structures observed after 18 h induction (**Fig. 3** **.B**)

The association of DGAT1 with LDs and the ER was previously demonstrated by Bouvier-Navé *et al*. [27]. DGAT2 localization to microsomes was deduced from *in vitro* assays by Zhou *et al.* [33], however its presence in other compartments cannot be excluded. Recent results by Kwiatkowska *et al.* tend to suggest multiple localizations of DGAT2 in germinated *A. thaliana* seeds [35]. These last experiments were based on the use of antibodies directed against human DGAT2, and the cross-reactivity between plant proteins and human antibodies needs to be confirmed. Taken together, these reports and our present findings appear to support the general model proposing that TAG synthesis occurs at the ER with LDs budding from the ER [34].

## Conclusion

We have described the enzymatic function and localization of *A. thaliana* DGAT2 and DGAT1 in the yeast quadruple mutant H1246. Functional complementation confirmed that DGAT2 has diacylglycerol acyltransferase activity. Differences observed at the level of FA composition suggest different substrate specificities for DGATs. Our study, together with previous reports from other teams, highlights the importance of tissue-specific factors and tRNA pools in regulating DGAT expression. Optimization of codon usage appears to be a valuable tool for increasing DGAT expression and subsequent lipid accumulation in yeast. Accumulation of squalene, a terpene with potential biotechnological applications, increased significantly by expression of Arabidopsis DGAT1. Overall, our results suggest that the nature of the DGAT expressed in yeast, is not only important for TAG accumulation, but also for the production of other lipids of interest.

## Supporting Information

Figure S1 **Native and optimized gene sequences used in this study.** Sequences were aligned using BioEdit [57].
(TIF)

Figure S2 **Identification of squalene in yeast total lipid fractions. (A)** Chromatogram of authentic squalene (upper panel, retention time 23.5 min) and of total lipids from DGAT1_opt_ expressing strain (lower panel) **(B)** Fragmentation spectra of authentic squalene (upper panel) and of the molecule eluting at 23.5 min (lower panel).
(TIF)

Figure S3 **TAG profiling of strains expressing DGAT.** Partial GC-FID chromatogram (ZB5-HT column) focused on separation of TAG compounds of DGAT expressing strains and H1246 transformed with the empty vector (control) lipid extracts.
(TIFF)
