# Supplementary figures and images for: Function and Localization of the Arabidopsis thaliana Diacylglycerol Acyltransferase DGAT2 Expressed in Yeast
Source: PLoS One. 2014 Mar 24;9(3):e92237. doi: 10.1371/journal.pone.0092237 (PMC3963872; doi:10.1371/journal.pone.0092237)

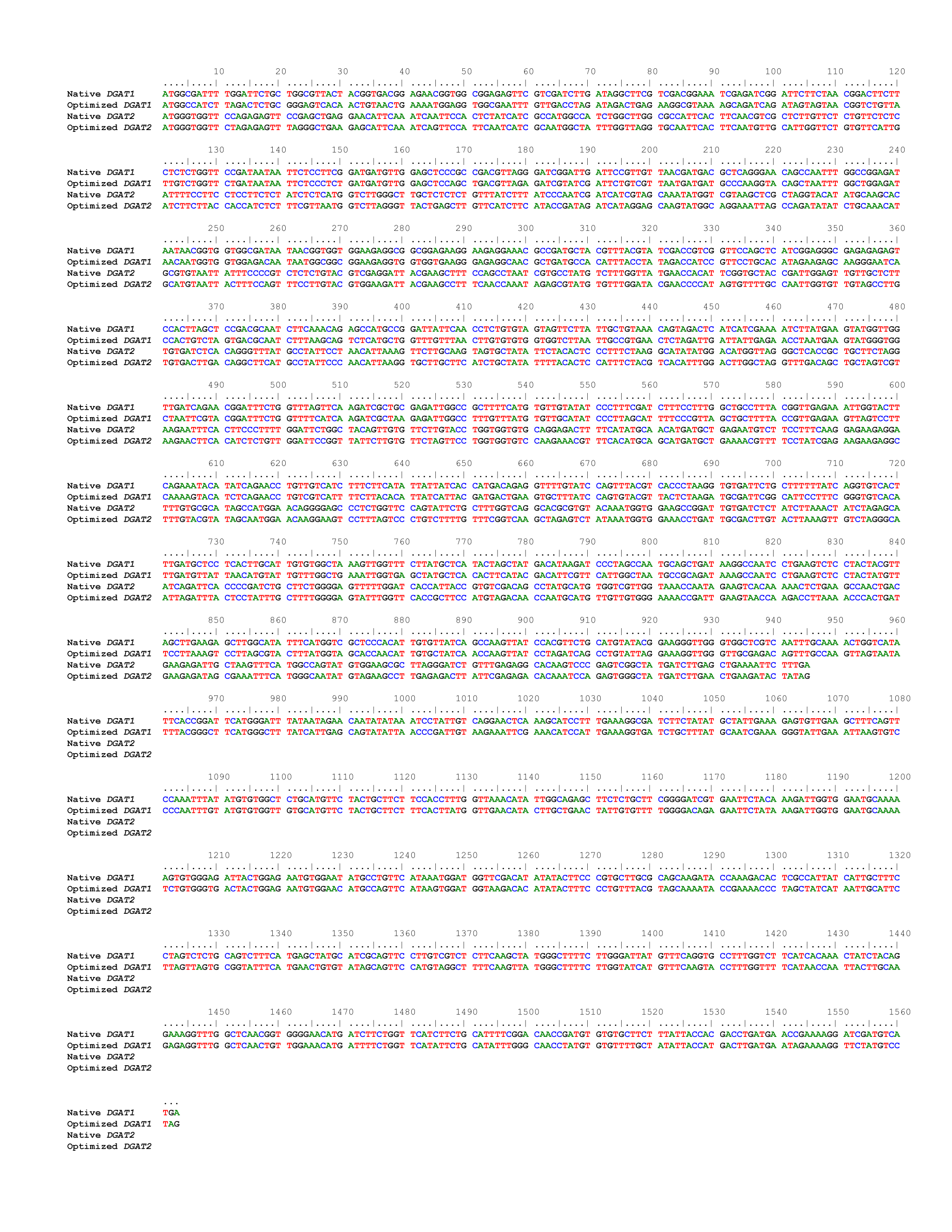

Supplement: Figure S1 — Native and optimized gene sequences used in this study. Sequences were aligned using BioEdit [57]. (TIF) [file pone.0092237.s001.tif]

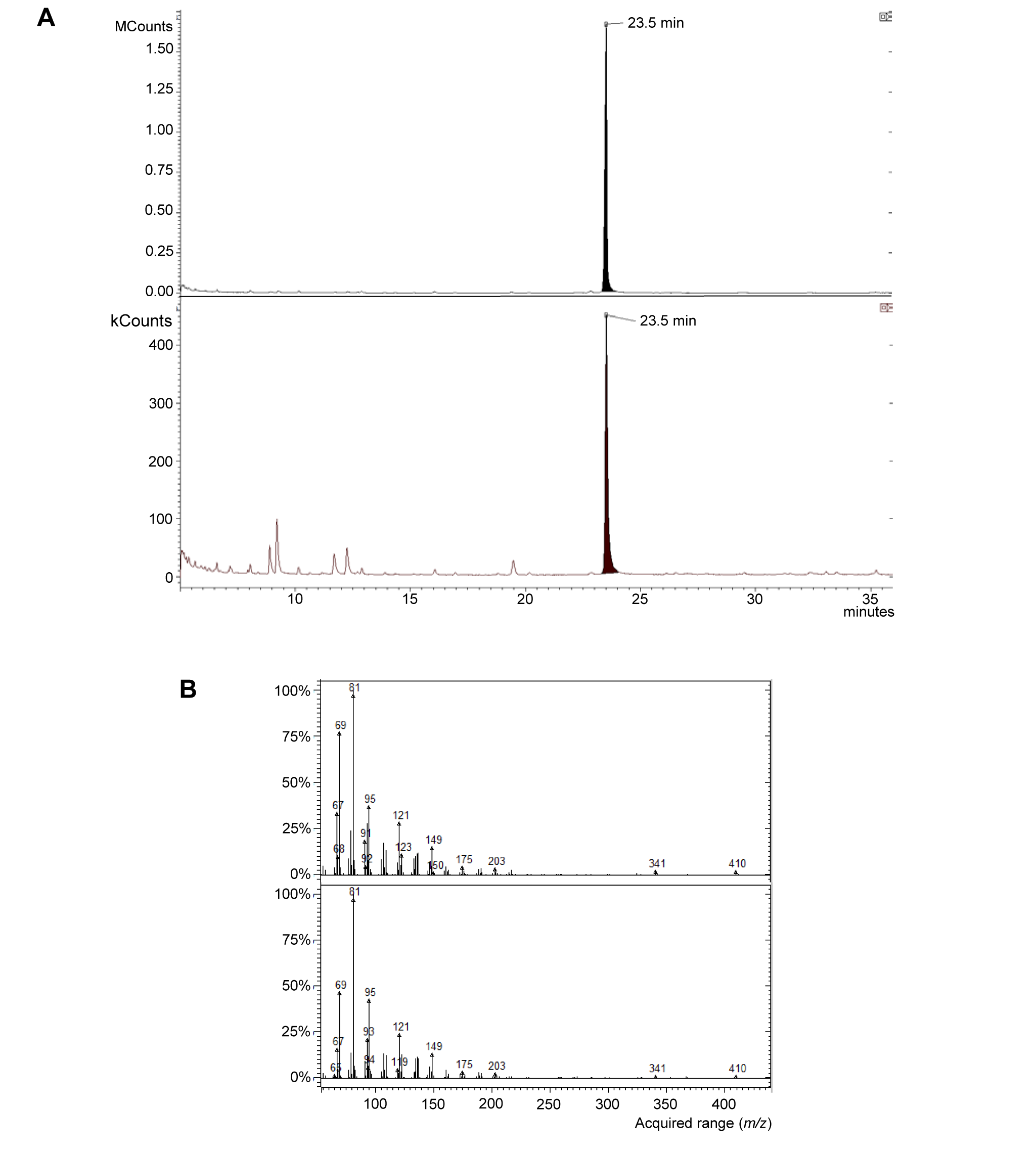

Supplement: Figure S2 — Identification of squalene in yeast total lipid fractions. (A) Chromatogram of authentic squalene (upper panel, retention time 23.5 min) and of total lipids from DGAT1opt expressing strain (lower panel) (B) Fragmentation spectra of authentic squalene (upper panel) and of the molecule eluting at 23.5 min (lower panel). (TIF) [file pone.0092237.s002.tif]

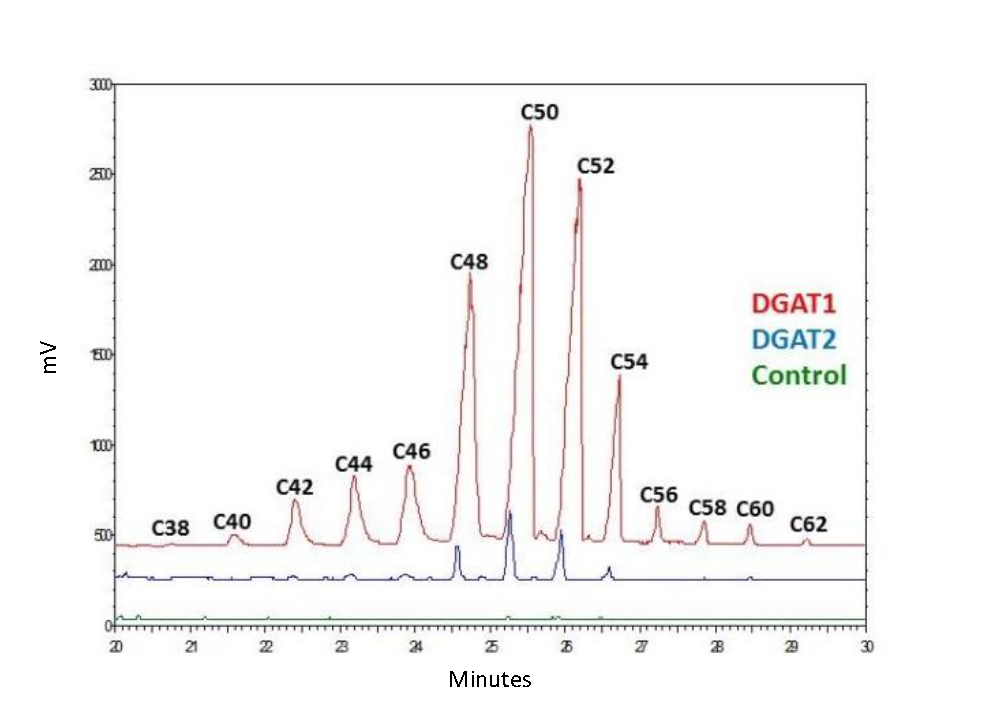

Supplement: Figure S3 — TAG profiling of strains expressing DGAT. Partial GC-FID chromatogram (ZB5-HT column) focused on separation of TAG compounds of DGAT expressing strains and H1246 transformed with the empty vector (control) lipid extracts. (TIFF) [file pone.0092237.s003.tiff]
